# Assessing the Functional Heterogeneity of Monocytes in Human Septic Shock: a Proof-of-Concept Microfluidic Assay of TNFα Secretion

**DOI:** 10.3389/fimmu.2021.686111

**Published:** 2021-07-05

**Authors:** Jean-François Llitjos, Yacine Bounab, Christophe Rousseau, Sophie Dixneuf, Blandine Rimbault, Jean-Daniel Chiche, Julien Textoris, Frédéric Pène, Christophe Védrine

**Affiliations:** ^1^ Institut Cochin, U1016, CNRS UMR8104, Paris, France; ^2^ UMR-S8104, Université de Paris, Paris, France; ^3^ Médecine Intensive et Réanimation, Hôpital Cochin, Assistance Publique – Hôpitaux de Paris APHP-CUP, Paris, France; ^4^ Bioassays, Microsystems and Optical Engineering, BIOASTER, Lyon, France; ^5^ EA7426 “Pathophysiology of Injury-induced Immunosuppression”, Université Claude Bernard Lyon-1 - HCL- BioMérieux, Lyon, France; ^6^ Medical Diagnostic Discovery Department (MD3), bioMérieux S.A., Lyon, France; ^7^ Anesthesiology and Critical Care Medicine, HCL, Lyon, France

**Keywords:** septic shock, immune suppression, monocyte, microfluidic, tolerance

## Abstract

**Objective:**

The development of advanced single-cell technologies to decipher inter-cellular heterogeneity has enabled the dynamic assessment of individual cells behavior over time, overcoming the limitation of traditional assays. Here, we evaluated the feasibility of an advanced microfluidic assay combined to fluorescence microscopy to address the behavior of circulating monocytes from septic shock patients.

**Methods:**

Seven septic shock patients and ten healthy volunteers were enrolled in the study. Using the proposed microfluidic assay we investigated the production over time of LPS-elicited TNFα by single monocytes encapsulated within droplets. Cellular endocytic activity was assessed by internalization of magnetic nanoparticles. Besides, we assessed HLA-DR membrane expression and LPS-induced TNFα production in monocytes through classical flow cytometry assays.

**Results:**

Consistent with the flow cytometry results, the total number of TNFα molecules secreted by encapsulated single monocytes was significantly decreased in septic shock patients compared to healthy donors. TNFα production was dampened as soon as 30 and 60 minutes after LPS stimulation in monocytes from septic patients. Furthermore, the microfluidic assay revealed heterogeneous individual behavior of monocytes from septic shock patients. Of note, monocytes from both healthy donors and patients exhibited similar phagocytic activities over time.

**Conclusion:**

The microfluidic assay highlights the functional heterogeneity of monocytes, and provides in-depth resolution in assessing the hallmark monocyte deactivation encountered in post-septic immunosuppression.

## Introduction

Sepsis is a complex life-threatening syndrome caused by a dysregulated host response to infection (1). The primary inflammatory response is often followed by a complex and protracted immunosuppressive response affecting both the innate and adaptive components of immunity. The hallmark features of sepsis-induced immunosuppression includes monocyte deactivation defined by reduced expression of HLA-DR, dampened pro-inflammatory cytokine production towards *ex vivo* lipopolysaccharide (LPS) stimulation, lymphocyte apoptosis, expansion of regulatory T-cells, apoptosis and defective antigen presentation of dendritic cells (2, 3). Septic shock is the most severe presentation and is associated with a particular risk of intensive care unit (ICU)-acquired infections and an eventual 40-percent mortality rate. The persistence of monocyte deactivation in septic patients, most commonly identified by decreased membrane HLA-DR expression, has been firmly associated with increased susceptibility to ICU-acquired infections and increased mortality, and may represent an actionable therapeutic target in this setting (4).

Appropriate characterization of sepsis-induced immune dysfunctions is key to the development of innovative therapeutic strategies. Routine assays provide average measurements of cell subsets but are poorly discriminant since unable to capture the behavior of individual cells. For instance, ELISA measurement of TNFα release by peripheral blood mononuclear cells (PBMC) after *ex vivo* LPS stimulation is a bulk measurement that overlooks the underlying complexity of different cell subsets. The development of advanced technologies such as RNA-sequencing or high-parameter mass cytometry now provides insights in deciphering inter-cellular heterogeneity at a single-cell resolution but shows limitations in addressing dynamic assessments in individual cells over time (5, 6). Furthermore enzyme-linked immunospot (ELISPOT), commonly used to detect proteins secreted from individual cells, is limited to endpoint measurements and is poorly quantitative. We have developed an ultrasensitive in-droplet immunoassay, in which an advanced microfluidic assay combined to fluorescence microscopy allows addressing the time course of LPS-elicited TNFα production by single monocytes encapsulated within droplets (7). Thousands of single in-droplet cells can thereby be monitored at the same time. This miniaturized assay enables simultaneous and dynamic measurements of protein secretion rate, cell surface marker, viability and phagocytic activity. In order to better characterize monocyte deactivation in sepsis, we applied this technique to blood samples obtained from septic shock patients as well as from healthy volunteers as controls.

## Methods

### Patients and Healthy Subjects

This prospective observational study was conducted from October 2018 to November 2019. Patients with septic shock were hospitalized in a medical ICU of a tertiary urban hospital. Septic shock was defined as microbiologically-proven or clinically-suspected infection associated with acute circulatory failure requiring vasopressors despite adequate fluid filling and serum lactate level ≥ 2 mmol/L. Patients were excluded if under 18 years of age, pregnancy, prior immunosuppression including leucopenia < 1 G/L related to underlying bone marrow disease or cytostatic chemotherapy, prior hospitalization during the last month or end-of-life decisions. We aimed at screening consecutive eligible patients, although some patients finally could not be enrolled due to logistic and technical issues in microfluidic assays. Blood samples (7 mL on heparin-coated vial and 7 mL on EDTA-coated vial) were collected within 72 hours from initiation of vasopressors. The following parameters were collected: demographics, source and microbiological documentation of infection, severity as assessed by the Simplified Acute Physiology Score 2 (SAPS2; range 0–194) and the Sepsis-related Organ Failure Assessment (SOFA; range 0–24) (8, 9). All experiments were performed in accordance with French ethical guidelines and regulations. The protocol for septic patients was approved by the institutional review board (Comité de Protection des Personnes du Sud-Ouest et Outre-Mer IV, #2017-A01134-49). Signed informed consent was obtained from patients or next of kin. Blood samples from healthy volunteers were obtained after informed consent from the ICAReB platform (Investigation Clinique et Accès aux Ressources Biologiques, Pasteur Institute, Paris) as part of the DIAGMICOLL or CoSImmGEn protocol (N° CORC: 2008-16 and 201-06), approved by the institutional review board (Comité de Protection des Personnes Ile-de-France). All tubes from patients and healthy subjects were anonymized at the time of blood sampling.

### Flow Cytometry

Membrane expression of HLA-DR onto monocytes and LPS-induced intracellular expression of TNFα were assessed by flow cytometry. Fluorescent antibodies anti-HLA-DR PE, Anti-CD14 PerCP-Cy5.5 and anti-TNFα PE were purchased from QuantiBRITE, Becton-Dickinson. Briefly, 25 µl of whole blood was mixed with 10 µl of antibody mix (anti-HLA-DR PE, Anti-CD14 PerCP-Cy5.5) and incubated at room temperature for 30 minutes in the dark. Red blood cells were lysed by adding 250 µl of diluted FACS lysing solution (Becton-Dickinson). The pellet was resuspended in 500µl of fixative solution (PBS supplemented with 1% PFA). Acquisition was immediately carried out on Accuri C6 cytometer. For intra-cellular TNFα staining, 1 mL whole blood was mixed with 5 μg/mL Brefeldin A (Biolegend) with or without 1μg LPS (LPS ultrapure EK12, InVivoGen) and incubated at 37°C for three hours. Cells were stained with anti-TNFα antibody in phosphate-buffered solution supplemented with 2% fetal calf serum, 2 mM EDTA and BD fix/perm solution (Becton-Dickinson). Monocytes were first gated on CD14 expression, to reach a count of at least 1500 CD14^+^ cells. Surface mHLA-DR expression was then measured (monoparametric histogram) with mean fluorescence intensity (MFI) related to the whole monocyte population. Fluorescence-minus-one control (FMO) controls were used to determine HLA-DR- and TNFα-positive monocytes.

### Droplet Production Chip and Droplet Imaging Chamber

The fabrication protocol of the microfluidic chip has been previously described by Mazutis and colleagues (10). Briefly, a master mold containing the desired pattern is used for producing the microfluidic geometry on poly(diméthylsiloxane) (PDMS) slabs. The droplet generator consists of a flow-focusing junction and produces highly monodisperse and stable aqueous droplets in inert fluorinated oil (HFE-7500, 3M) supplemented with 2% nonionic perfluoro-polyether fluoro-surfactant (Ran Biotechnologies). The device configuration consists in three inlets to bring respectively the carrier oil, the cell suspension and the assay reagents. The produced droplets are collected at the outlet of the microfluidic chip prior injection in a static microfluidic chamber made of two glass slides sealed using a 48 µm thick double-sided adhesive tape, which thus guarantees a single layer of droplets (diameter ~ 46 µm).

### Nanoparticles Functionalization and Use

A boronic acid-based coupling strategy was used for precise orientation of anti-TNFα antibodies (Antibody TNF5, Mabtech) on the surface of 300 nm magnetic nanoparticles (Ademtech, cat. no 2131). 3-aminophenylboronic acid molecules (Sigma) were grafted on the surface of carboxylated beads to act as an affinity head-group for covalent and site-specific IgG attachment. Functionalized nanoparticles were validated and calibrated in microfluidics conditions. Briefly, dose response experiments were performed using recombinant TNFα protein (BioLegend). Resulting calibration data were used to convert nanoparticle fluorescence signal into a number of detected TNFα molecules. The encapsulation of 170 nanoparticles per droplet resulted in the formation of a single homogenous bead-line (in the left part of the droplet) after application of a permanent magnetic field (strength B= 0.22 Tesla).

### Single Monocyte Encapsulation

Cell and reagents were prepared and encapsulated in droplet as previously described (7). Briefly, monocytes from healthy donors or septic shock patients were enriched with an EasySep Direct Human Monocyte Isolation Kit (STEMCELL Technology). Monocytes were encapsulated into 50-pL droplets together with cell culture medium RPMI1640 Medium GlutaMAX (Gibco) supplemented with 10% (vol/vol) heat-inactivated fetal bovine serum (HyClone), 5% (vol/vol) penicillin-streptomycin (Life Technologies), and 20 mM HEPES (Dominique Dutscher), as well as with anti-TNFα coated-nanobeads, phycoerythrin-labeled anti-TNFα detection antibody (cA2 Clone, Miltenyi Biotec), LPS (Sigma-Aldrich) and cell survival probes to detect mitochondrial membrane potential (MitoView633, Biotium) and to measure caspase-3 activity (NucView488, Biotium). The resulting emulsion was transferred into the microfluidic-imaging chamber as described above. After filling, the chamber was mounted onto a fluorescence microscope for time-lapse kinetic measurement.

The number of cells encapsulated in droplets follows a Poisson distribution. The statistical study focused on droplets that actually contained one single monocyte, which averages 10% of droplets for healthy donors and patients. Although nanoparticles distribution in droplets is binomial, every droplet contained approximately 170±7 nanoparticles. Finally, molecular reagents (phycoerythrin-labeled anti-TNFα detection antibody, LPS and cell survival probes) were homogenously distributed in all droplets. Cells were stimulated by LPS at 0.25µg/mL in droplets. LPS stimulation somewhat differed between microfluidic and cytometric assays in terms of concentrations within cells’ environment (whole blood versus single monocyte in a droplet).

### Automated Image Analysis and Processing

Time-lapse imaging was performed using a thermalized motorized Nikon inverted microscope and x10 magnification. For each experiment, images were acquired in three fluorescence channels (excitation/emission: 482/520 nm, 531/593 nm, 628/692 nm) every 30 min over 3 hours at the temperature of 37°C, and then analyzed by R software using an automated Matlab script for extracting time-lapse fluorescence levels.

### Endocytosis Monitoring

Magnetic nanoparticles can bind to the cell surface and be internalized through cellular endocytic activity. Our platform has the capacity to track monocytes that internalize nanoparticles. Briefly, the location of the cell relatively to the location of the beadline (i.e. the aligned magnetic nanobeads) was used to assess an effective endocytosis of the beadline by the cell.

### Image Processing

A Matlab-based software detects and tracks droplets in time-lapse image sequences and monitor the status of their content throughout the kinetics. For each field, the analysis procedure can be summarized in three steps: (i) detect contours of the droplets and intra-droplets beadline and cells at t0, (ii) sort the droplets according to the number of cells they contain and initial cell status (alive or dead), and (iii) track the droplets of interest (i.e. strictly containing a single live cell) for monitoring beadline fluorescence and cell status evolution at all time. The software implements the Matlab Image processing toolbox as well as the VLFeat open source library. Software output is a spreadsheet containing localization, morphological and intensity parameters for different intra-droplet objects over time.

### Statistics

Continuous variables were expressed as mean ± standard deviation. Biological results were compared between patients and healthy subjects using Student’s t-test. p<0.05 or p<0.001 indicated statistically significant differences.

## Results

### Patients and Control Subjects

Seven septic shock patients and 10 healthy donors were enrolled in the study. Half of healthy donors were males and their age was 46 years (± 9.2). Individual data of septic shock patients are given in **Table 1**. All patients were sampled within the first 48 hours after diagnosis of septic shock [time from initiation of norepinephrine to sampling 22 hours (± 13)].

**Table 1:** Main characteristics of patients with septic shock.

Patients’ Identification	Gender	Age, years	Source of infection	Admission SOFA	Admission SAPS2	First lactate, mmol/L	WBC count, G/L	Monocytes count, G/L
**# 1**	Male	43	Lung	7	77	9.3	3.5	0
**# 2**	Male	70	Lung	21	87	8.6	1.8	0.05
**# 3**	Male	59	Lung	9	54	3.6	34.2	1.12
**# 4**	Male	86	Urinary tract	2	45	2.6	40.2	0.96
**# 5**	Male	31	Lung	8	76	2	16.9	0.86
**# 6**	Female	73	Meningeal	2	39	4.4	11.9	0.5
**# 7**	Male	80	Lung	4	37	2.3	14.3	0.8

Age is expressed in years, SOFA, Sequential Organ Failure Assessment; SAPSII, simplified acute physiology score II; WBC, white blood cells.

### Flow Cytometry Assessment of Monocyte Deactivation

**Figure 1:**
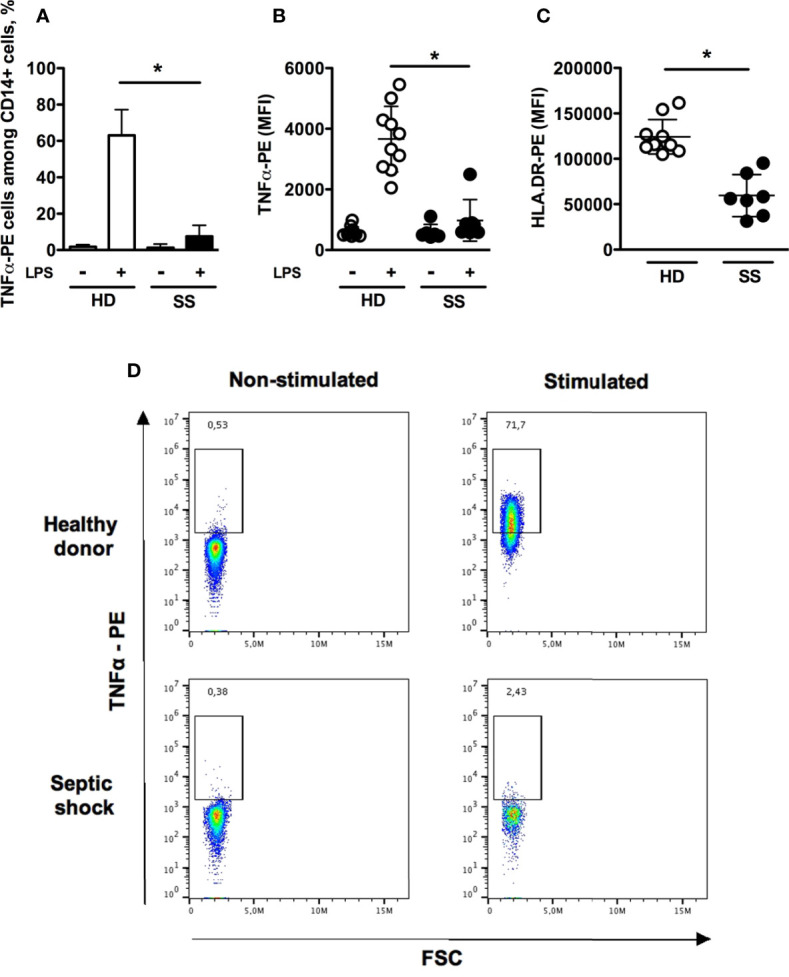
Flow cytometry assessment of monocyte deactivation. Monocytes were stimulated in *vitro* with 1 μg/mL LPS for 180 minutes. **(A)** Proportions of TNFα-positive monocytes without or with LPS stimulation in healthy donors (n = 10) and septic shock patients (n=7). **(B, C)** TNFα intracellular staining (with or without LPS stimulation) and HLA-DR membrane expression. **(D)** Representative dot-plots of intracellular production of TNFα. Data are expressed as mean and standard deviation. *p < 0.001.

The hallmark features of monocyte deactivation, namely decreased HLA-DR membrane expression and impaired TNFα production in response to LPS stimulation, were assessed for circulating monocytes from septic shock patients and compared to those from healthy donors. TNFα-secreting cells accounted for 7.5 ± 6% and 63 ± 14.1% of monocytes in patients and healthy donors, respectively (**Figures 1A, D**). In addition both the intracellular TNFα expression and HLA-DR surface expression were significantly lower in septic shock patients as compared to healthy donors (**Figures 1B, C**).

### Single-Cell Dynamic Microfluidic Analysis

**Figure 2:**
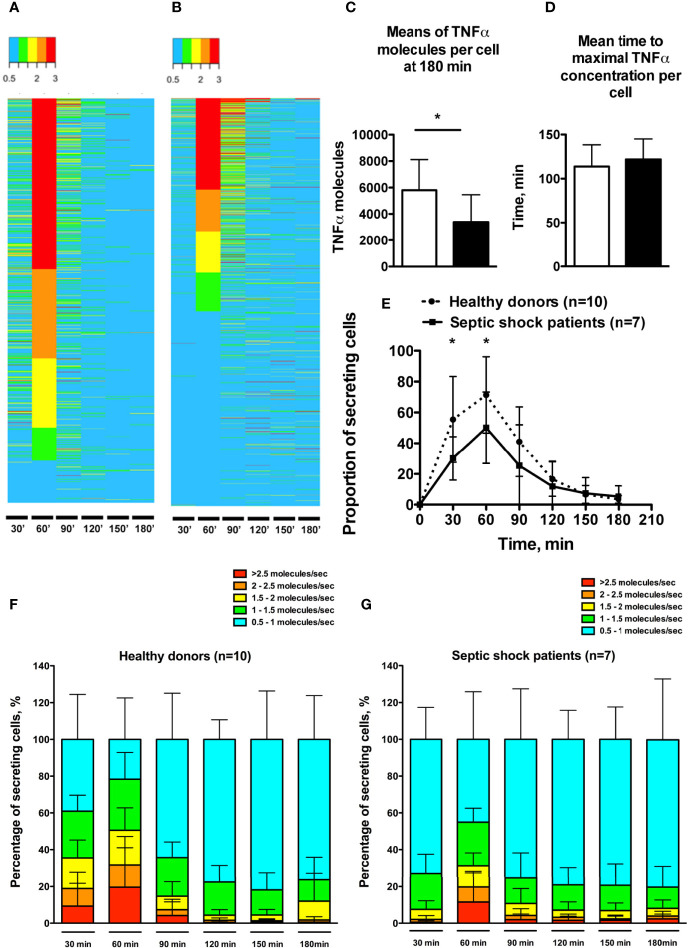
Single-cell dynamic microfluidic analysis of monocyte TNFα secretion under LPS stimulation. **(A, B)** Heatmaps expressing TNFα secretion rate over time in one healthy donor (988 cells) **(A)** and one septic shock patient (1021 cells) (**B**). Cells were clustered based on TNFα secretion rate at 60 min. Secretion rate is expressed in TNFα molecules per second. **(C)** Means of maximal amount of TNFα molecules in cells. **(D)** Mean time to reach the maximal concentration of TNFα in each cell. **(E)** Proportion of monocytes secreting TNFα (molecules/sec) over time after LPS stimulation. **(F, G)** Proportions of secreting monocytes clustered by TNFα secretion rates over time in healthy donors **(F)** and septic shock patients **(G)**. *p < 0.05.

**Figure 3:**
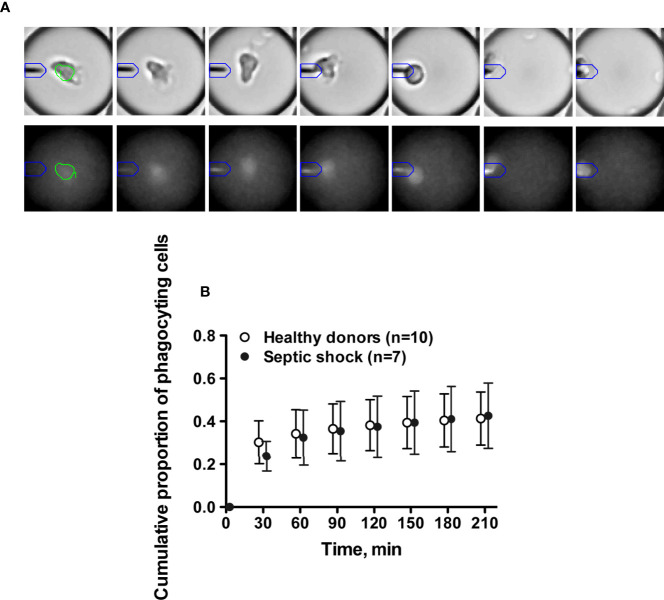
Single cell dynamic microfluidic monitoring of monocyte phagocytosis. **(A)** Kinetics of phagocytosis activity of one monocyte starting at 90 min. Brightfield images (upper panel) illustrate cell migration toward the beadline (from center to the left of the droplet). Fluorescence images (lower panel) show the impact of phagocytosis on the beadline fluorescence signal (fuzzy fluorescence signal at 180 min due to the loss of beadline integrity). **(B)** Cumulative proportions of monocytes with phagocytosis of magnetic beadline over 180 min after LPS stimulation.

The microfluidic dynamic assay (**Supplementary Figure 1**) allows a continuous measurement over time of molecules that are secreted by single cells (**Figures 2A, B**). Consistent with the flow cytometry results, the total number of secreted TNFα molecules by encapsulated single cells was significantly lower in septic shock patients compared to healthy donors (3390 ± 2071 *vs.* 5807 ± 2309 molecules, p=0.04) (**Figure 2C**). The time to reach total TNFα release was not significantly different between healthy donors and septic shock patients (**Figure 2D**). However, TNFα production by monocytes from septic shock patients was lower through the decreased proportion of secreting monocytes at early time points of 30 minutes and 60 minutes after LPS stimulation (**Figure 2E**). Of note, monocytes from healthy donors and from septic patients exhibited similar phagocytic activities over time (**Figure 3**).

The microfluidic assay revealed heterogeneous individual behavior of monocytes. Thus monocytes from healthy donors displayed TNFα secretion rates ranging from 0.5 to >2.5 molecules/sec (**Figure 2F**). In septic shock patients the proportion of cells with secretion rate > 1.5 molecule/sec was decreased at early time points (30 min and 60 min) (**Figure 2G**). Furthermore, the septic shock condition likely smoothed the heterogeneity in TNFα secretion rate by monocytes, as suggested by the increased proportions of cells displaying slow TNFα secreting rates at each time point.

## Discussion

We identified different patterns of cytokine responsiveness to *in vitro* LPS stimulation across monocytes from septic shock patients, which were characterized by slowdown in TNFα secretion and lower cell response diversity restricted to slow TNFα-secreting cells. In contrast, it is noteworthy that monocytes’ phagocytic capacities were preserved.

Monocytes represent a heterogeneous population of cells differing in morphology, gene expression, surface markers and functions (11). Based on the membrane surface expression of CD14 and CD16, monocytes are classically distributed into three subsets of different functions. The classical CD14^+++^CD16^-^ subset accounts for up to 80% of circulating monocytes and harbors potent phagocytic functions. Intermediate CD14^+^CD16^+^ monocytes display both phagocytic and cytokine secretion functions and increase under inflammatory conditions. Non-classical CD14^+^CD16^+++^ monocytes have a patrolling role (12). An infectious insult can induce early and transient monocytopenia within all three subsets (13), ascribed to increased adherence to vessel walls and cell recruitment to inflammatory sites, and restored by monocytes released from bone marrow (14). Besides, functional defects of circulating monocytes have been associated with outcomes in septic shock. HLA-DR expression onto monocytes is currently viewed as the soundest biomarker of post-septic immune suppression, associated with increased risk of ICU-acquired infections and increased mortality (2, 4). Furthermore, a few studies have suggested its potential in a personalized medicine approach to identify patients likely to benefit from adjuvant immune-enhancing treatments, interferon gamma or granulocyte-monocyte colony-stimulating factor, to reverse monocyte deactivation (15, 16).

With regard to its pivotal role in antigen-presentation and antibacterial response, expression level of human leucocyte antigen-D related (mHLA-DR) in circulating monocytes has gained considerable interest over the past decades in identifying altered immune status among severe ICU patients. Even if preliminary studies report interesting results of mRNA expression levels of HLA-DR in whole blood, robustness and reproducibility of flow cytometry support its use in determining mHLA-DR expression (17). These technical considerations raise two important points of discussion. First, transposal of functional alterations observed in circulating cells to their resident counterparts is questionable with respect to the compartmentalization of inflammatory responses and the functional specialization of tissular macrophages. Second, characterization of monocytes in humans is based on summed measurements within one cell subset, assuming that individual cells harbor similar functional behavior, but without any assessment in reaction speed nor in sustainability of cell responses. Thanks to an advanced microfluidic assay, we now challenge this global view by evidencing the dynamic functional heterogeneity of monocytes.

Our study revealed that the decrease in TNFα secretion rate in septic patients was mostly observed at early time points following LPS stimulation. Monocytes from septic shock exhibited a characteristic behavior of cytokine hyporesponsiveness while preserved uptake functions, consistent with a “reprogrammed” rather than “exhausted” phenotype. Accordingly, transcriptomic assays in LPS-challenged peripheral blood mononuclear cells from patients facing Gram-negative infections retrieved endotoxin tolerance while enhanced anti-microbial activity and tissue repair (18). The transcription factor hypoxia-inducible factor-1α (HIF-1α) has been involved in the transition from pro-inflammatory to anti-inflammatory monocytes/macrophages (19).

We also observed an important heterogeneity in TNFα secretion rate of monocytes in septic shock patients. To our knowledge, only a few studies have addressed the intercellular heterogeneity in sepsis. Using single-cell RNA-seq analysis, it has been reported that *Salmonella* infected macrophages display high cell-to-cell differences in gene expression (5). Similarly, bone marrow-derived dendritic cells exhibit extensive heterogeneity in their transcriptomic response to various bacterial ligands (20). Considering the absence of dynamic monitoring, these approaches cannot distinguish between heterogeneous responses related to various stages of maturation and the prior coexistence of different immune cell subsets committed to differential responses to infection. Using dynamic single-cell analysis and a homogeneous immune stimulation, our results suggest that cell-to-cell variations preferentially reflect the existence of distinct pre-existent subsets rather than different stages of maturation.

This study has several limitations. First, this exploratory study was limited to a small number of patients and controls. However, it reached its primary goal to provide a more accurate view of monocytes’ behavior in a characteristic clinical condition underlay by acute immune changes. It was definitely not designed to establish correlations between TNFα secretion profiles and relevant clinical outcomes. Second, the cytokine secretion pattern was limited to the pro-inflammatory cytokine TNFα, and we did not address the production of alternative mediators such as the prototypic anti-inflammatory cytokine IL-10. Third, monocytes were only identified through CD14 expression without additional CD16 staining to identify specific subsets. Fourth, single-cell dynamic microfluidic analysis is time-consuming and cannot be considered in routine analysis yet.

## Conclusion

Our results add a new layer of complexity in sepsis-induced immune alterations by highlighting the functional heterogeneity of monocytes. The impact on clinical outcomes and how pharmacological interventions to reverse monocyte deactivation may target the different functional subsets are unanswered questions that deserve further investigations using high-resolution microfluidic assay.

## Data Availability Statement

The raw data supporting the conclusions of this article will be made available by the authors, without undue reservation.

## Ethics Statement

The studies involving human participants were reviewed and approved by Comité de Protection des Personnes du Sud-Ouest et Outre-Mer IV. The patients/participants provided their written informed consent to participate in this study.

## Author Contributions

J-FL, YB, JT, FP, and CV designed the study. J-FL, J-DC, and FP Enrolled Patients. J-FL and CR performed cytometry experiments. YB performed microfluidic experiments. SD and BR performed statistical analysis. J-FL, FP, and CV drafted the manuscript. All authors contributed to the article and approved the submitted version.

## Funding

The project was funded by a consortium: bioMérieux, SANOFI, GlaxoSmithKline, Ecole Supeérieure de Physique Chimie Industrielles de la Villette Paris–PSL Research University, the University Hospital Hospices Civils de Lyon and the microbiology technological institute BIOASTER. The funders were not involved in the study design, collection, analysis, interpretation of data, the writing of this article or the decision to submit it for publication. This research project has received funding from the French Government through the “Investissement d’Avenir” program (grant n°ANR-10-AIRT-03).

## Conflict of Interest

JT is an employee of bioMérieux, S.A. YB and CV are inventors on a patent application based on certain ideas described in this manuscript and may receive financial compensation *via* their employer’s rewards to inventors’ scheme.

The remaining authors declare that the research was conducted in the absence of any commercial or financial relationships that could be construed as a potential conflict of interest.

## Supplementary Material

Supplementary Figure 1 Schematic overview of cytokine secretion assay in droplet. **(A)** Human monocytes were isolated from human blood and stained with a fluorescent anti-CD14 antibody. **(B)** Nanoparticle functionalization and bioassay preparation. The bioassay comprises magnetic nanobeads (300nm diameter) functionalized with anti-TNFα antibodies, fluorescent-labeled anti-TNFα detection antibodies, live cell probes (red dots), dead cell probes (green dots) and LPS ligand (black dots). **(C)** Co-encapsulation of monocytes and assay reagents into 50–pL droplets in a microfluidic device. **(D)** Emulsion transfer into a glass chamber to form a monolayer array of droplets. **(E)** Time-lapse imaging of droplets using an epifluorescence microscope (37°C) and automated image analysis using in-house droplet-tracking software.
