# Supplementary figures and images for: Assessing the Functional Heterogeneity of Monocytes in Human Septic Shock: a Proof-of-Concept Microfluidic Assay of TNFα Secretion
Source: Front Immunol. 2021 Jul 5;12:686111. doi: 10.3389/fimmu.2021.686111 (PMC8288100; doi:10.3389/fimmu.2021.686111)

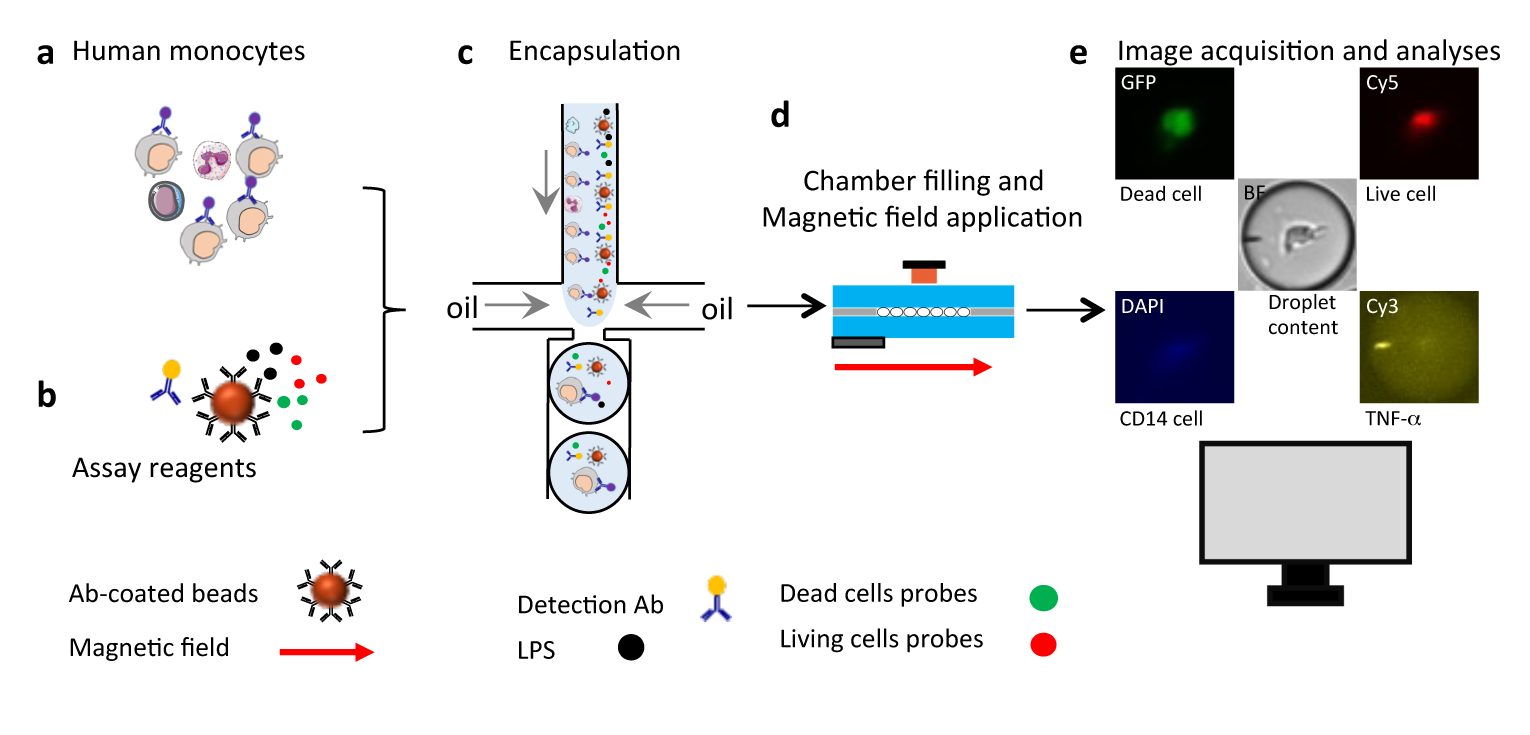

Supplement: Supplementary Figure 1 — Schematic overview of cytokine secretion assay in droplet. (A) Human monocytes were isolated from human blood and stained with a fluorescent anti-CD14 antibody. (B) Nanoparticle functionalization and bioassay preparation. The bioassay comprises magnetic nanobeads (300nm diameter) functionalized with anti-TNFα antibodies, fluorescent-labeled anti-TNFα detection antibodies, live cell probes (red dots), dead cell probes (green dots) and LPS ligand (black dots). (C) Co-encapsulation of monocytes and assay reagents into 50–pL droplets in a microfluidic device. (D) Emulsion transfer into a glass chamber to form a monolayer array of droplets. (E) Time-lapse imaging of droplets using an epifluorescence microscope (37°C) and automated image analysis using in-house droplet-tracking software. [file Image_1.tif]
